# Mental Health and Psychological Constructs Related to Performance in Professional, Semi-Professional, and Competitive Adolescent Women’s Soccer Players: A Scoping Review

**DOI:** 10.3390/sports14090390

**Published:** 2026-09-03

**Authors:** Aura D. Montenegro-Bonilla, Boryi A. Becerra-Patiño, Jorge Olivares-Arancibia, Julio Plaza-Díaz, Daniel Rojas-Valverde, Rodrigo Yañez-Sepúlveda, José Francisco López-Gil

**Affiliations:** 1 Faculty of Physical Education, National Pedagogical University, Bogota 480100, Colombia; admontenegrob@upn.edu.co (A.D.M.-B.); babecerrap@pedagogica.edu.co (B.A.B.-P.); 2 Doctoral Program in Physical Activity and Sport Sciences, University of Murcia, 30720 San Javier, Spain; 3 AFySE Group, Research in Physical Activity and School Health, School of Physical Education, Faculty of Education, Universidad de las Américas, Santiago 7500975, Chile; jorge.olivares.ar@gmail.com; 4 School of Health Sciences, Universidad Internacional de La Rioja, 26006 Logroño, Spain; julioramon.plaza@urv.cat; 5 Departament de Bioquímica i Biotecnologia, ANUT-DSM (Alimentació, Nutrició Desenvolupament i Salut Mental), Universitat Rovira i Virgili, 43201 Reus, Spain; 6 Institut de Recerca Biomèdica Catalunya Sud, Hospital Universitari Sant Joan de Reus, 43204 Reus, Spain; 7 Biomedical Research Networking Center for Physiopathology of Obesity and Nutrition (CIBERObn), Institute of Health Carlos III, 28029 Madrid, Spain; 8 Centro de Investigación, Desarrollo e Innovación en Salud y Deporte (CIDISAD), Escuela de Ciencias del Movimiento Humano y Calidad de Vida, Universidad Nacional, Heredia 86-3000, Costa Rica; drojasv@hotmail.com; 9 Clínica de Lesiones Deportivas (Rehab & Readapt), Escuela de Ciencias del Movimiento Humano y Calidad de Vida, Universidad Nacional, Heredia 86-3000, Costa Rica; 10 Faculty of Education and Social Sciences, Universidad Andres Bello, Viña del Mar 2520000, Chile; rodrigo.yanez.s@unab.cl; 11 Department of Health Research, Icen Cognis, 38370 La Matanza de Acentejo, Spain; 12 School of Medicine, Universidad Espíritu Santo, Samborondón 092301, Ecuador; 13 Faculty of Health Sciences, Universidad Tecnológica Atlántico Mediterráneo—UTAMED, 29590 Málaga, Spain

**Keywords:** women’s soccer, female soccer players, women’s football, mental health, psychological factors, scoping review, PRISMA-ScR, resilience

## Abstract

Background: Mental health has become a fundamental component of the sports world. Although previous studies have addressed psychological aspects of women’s soccer, no comprehensive review has mapped both mental health symptoms and adaptive psychological resources and psychophysiological markers in professional, semi-professional, and competitive adolescent women’s soccer. Objective: This scoping review aimed to map recent evidence from 2020 to 2025 on mental health in professional and semi-professional women’s soccer including competitive settings for adolescents, identifying key psychological constructs, assessment instruments, and risk and protective factors within the competitive sport context. Methods: Following the methodological guidelines of the Joanna Briggs Institute (JBI) and Preferred Reporting Items for Systematic Reviews and Meta-Analyses extension for scoping reviews (PRISMA-ScR), a systematic search was conducted in PubMed/MEDLINE, Scopus, and Web of Science. Thirteen empirical studies that explicitly assessed mental health dimensions in professional and semi-professional women’s soccer players were included. Results: The most frequently studied constructs were psychological stress, competitive anxiety, resilience, motivation, and well-being. The main risk factors included intense competitive demands, accumulated fatigue, and performance pressure. Predominant protective factors were individual resilience, social support, team cohesion, and self-confidence. The most used instruments were validated scales including the Brunel Mood Scale (BRUMS), Sport Anxiety Scale-2 (SAS-2), Competitive State Anxiety Inventory-2 Revised (CSAI-2R), Generalized Anxiety Disorder-7 (GAD-7), Sport Mental Health Assessment Tool-1 (SMHAT-1) and qualitative interviews. Resilience emerged as a central concept; although it was measured directly in only two studies, it emerged conceptually as a central theme in the evidence, and recent qualitative studies have highlighted the influence of the institutional environment and technical support. Conclusions: The available evidence which is heterogeneous and limited to 13 studies points to a growing body of research on mental health in women’s soccer, focused primarily on stress and resilience. However, significant gaps remain, including a lack of multi-season longitudinal studies tracking players across full competitive cycles, limited representation of Latin American and Asian contexts (one study each), the absence of position-specific analyses such as studies focused on goalkeepers and insufficient attention to positive well-being. Prospective and intervention-based studies are needed to develop a comprehensive understanding of mental health in elite sports and in competitive adolescent settings.

## 1. Introduction

Women’s soccer has undergone a substantial transformation over recent decades, particularly during the last thirty years, driven by increased development, visibility, and professionalization [1,2]. A systematic review examining major research areas in women’s soccer identified performance, motivation, and risk as the primary domains, often interconnected with injury prevention, physical performance, and sport participation motives [3]. These findings highlight the presence of research lines focused on psychological processes that contribute to understanding performance and its relationship with the professionalization of women’s soccer [4,5]. However, despite the exponential growth of the sport, there remains a paucity of studies clarifying how psychological factors influence players’ performance [6,7,8].

Some studies have reported associations between motivation, team cohesion, and improvements in well-being, often accompanied by reductions in anxiety levels [9,10]. Furthermore, psychophysiological evidence indicates that pre-competition heart rate variability decreases significantly during high-pressure matches, which in turn leads to an increase in anxiety levels before highly demanding matches [11]. These findings underscore the importance of investigating psychological factors that may influence the mental health of women’s soccer players. A bibliometric study on professional and recreational soccer players reported that the term “sport psychology” appears 124 times in soccer research overall, but only 23 times in studies focused on women’s soccer and just 6 times in professional women’s soccer [12]. Similarly, Ventaja Cruz et al. [13] concluded that research addressing well-being and sport motivation in women’s soccer remains limited, although scientific production in this area has increased since 2015. A systematic review examining psychological factors and their relationship with performance in women’s soccer found that higher-level players tended to exhibit superior executive function, greater mental toughness, higher responsibility, and lower levels of anxiety [7]. Nevertheless, despite the growing body of research on psychological factors, mental health remains an underrepresented domain in professional women’s soccer [14].

Prospective cohort studies with 12-month follow-up periods have reported that professional women’s soccer players experience psychological distress following surgical interventions, highlighting the importance of integrating sports medicine teams with professionals responsible for psychological support [15]. Other studies have identified severe injuries as a major determinant of mental health outcomes, often associated with sleep disturbances, addictive behaviors, and reduced well-being [16]. Meta-analytic evidence investigating mental health symptoms and disorders in elite athletes indicates prevalence rates of approximately 34% for anxiety and depression and 26% for psychological distress [17]. Moreover, it has been suggested that elite sports environments may expose athletes to up to 640 distinct stressors, many of which are linked to the demands of high-level competition and contribute to mental health vulnerability [17,18].

Mental health has become a critical component of the sport context. Women’s soccer are exposed to increasing performance demands, often experiencing stress, anxiety, anger, fatigue, and cognitive overload, which may also elevate the risk of physical injury [19,20]. These factors are closely associated with competitive pressure, performance expectations, and persistent structural inequalities in women’s sport, which can contribute to the development of mental health problems such as substance misuse, burnout, overtraining, and injury [21,22]. In professional sports settings, the accumulation of stressors (including career transitions such as retirement or dropout) may further exacerbate psychological vulnerability, particularly among high-performance athletes [23,24]. These stressors are intrinsically linked to the demands of high-performance sport and the pursuit of competitive success [25].

Recent evidence also indicates that the prevalence of mental health symptoms in a women’s soccer sample [26], psychological distress 49.5%, depression 44.7%, eating disorder symptoms 22.3%, and anxiety 20.4%. It should be noted that these figures derive from a single study with a specific semi-professional sample and should not be generalized to women’s soccer as a whole; rather, they illustrate that some studies report elevated symptom levels in specific samples. These findings reinforce the need to better understand the factors influencing emotional and psychological well-being in women’s soccer. Furthermore, mental health should not be considered as isolation from physical health. As highlighted by Reardon et al. [27], mental health disorders and symptoms can increase injury risk and hinder recovery, underscoring the importance of integrating psychological and physical health approaches within professional sport.

For the purposes of this review, mental health is defined in line with the World Health Organization’s conceptualization, as a state of psychological well-being that enables individuals to cope with stress, function productively, and realize their abilities, rather than the mere absence of clinical symptoms [28]. This review accordingly maps three related but distinct layers of evidence: (i) clinical or sub-clinical symptoms of psychopathology (e.g., depression, anxiety, disordered eating); (ii) psychological well-being and adaptive resources (e.g., resilience, mood, coping); and (iii) performance-related psychological constructs (e.g., motivation, mental toughness) only insofar as they intersect with players’ mental health. Studies based on performance constructs (e.g., motivational climate, personality traits) were considered eligible when they reported a relationship with at least one dimension of mental health or psychological well-being, and not when athletic performance was the only outcome assessed. This distinction is made explicit here to avoid conflating clinical and performance-psychology studies, which are frequently treated interchangeably in this field. This conceptual framework aligns with current models of athletes’ mental health, such as the International Olympic Committee’s framework on mental health in professional athletes and biopsychosocial models, which emphasize the interaction between physical, psychological, and sociocultural factors in determining athletes’ well-being [27]. Furthermore, this study aims to continue exploring the mental health of women’s athletes as an essential aspect of athletic development from the grassroots level to the professional level rather than as a mere secondary concern [29].

These findings highlight the need to systematically identify the main psychological factors affecting mental health in women’s soccer. A comprehensive synthesis of the available evidence can contribute to understanding the psychological constructs present in professional women’s soccer and support the development of targeted, interdisciplinary intervention models. Therefore, the objective of this scoping review is to map the available scientific evidence on mental health in professional and semi-professional women’s soccer including competitive settings for adolescents between 2020 and 2025, identifying the psychological constructs studied, the assessment instruments used, the associated risk and protective factors, and the existing gaps in the literature.

## 2. Materials and Methods

### 2.1. Study Design

This scoping review was conducted in accordance with the methodological guidelines of the Joanna Briggs Institute (JBI) for scoping reviews, which are internationally recognized for providing a structured, transparent, and reproducible framework for mapping evidence in emerging or conceptually heterogeneous fields [30]. This approach was considered appropriate given the conceptual heterogeneity in how mental health has been studied in women’s soccer players and the need to describe both the breadth and the characteristics of the available literature. The study was conducted following a predefined protocol, with no deviations from the original methodological plan.

### 2.2. Search Strategy

The search strategy was structured following the three-stage approach proposed by the JBI [30]. First, an initial exploratory search was conducted in MEDLINE/PubMed and Scopus to identify key articles and extract the most frequently used terms in titles, abstracts, and indexing. This process enabled the refinement of the Population-Concept-Context (PCC) framework and the identification of both controlled vocabulary (Medical Subject Headings [MeSH] terms) and free-text keywords. Second, a comprehensive search strategy was developed and adapted to each database: PubMed/MEDLINE, Scopus, and Web of Science. Search equations incorporated MeSH terms, synonyms, and Boolean operators (AND/OR), ensuring both sensitivity and specificity. The search was limited to studies published between January 2020 and December 2025, a period characterized by rapid development in women’s soccer and increased scientific attention to mental health following the Coronavirus Disease 2019 (COVID-19). The final database search was conducted on 10 December 2025. The conceptual equation was adapted to each database, as detailed below:

PubMed/MEDLINE: (“women soccer players” [tiab] OR “women’s soccer” [tiab] OR “female football players” [tiab] OR “women’s football” [tiab]) AND (“mental health” [tiab] OR “mental health” [MeSH Terms] OR anxiety[tiab] OR depression[tiab] OR stress[tiab] OR burnout[tiab] OR “mental fatigue” [tiab] OR wellbeing[tiab] OR “psychological well-being” [tiab] OR coping[tiab] OR resilience[tiab] OR “mental toughness” [tiab]).

Scopus: TITLE-ABS-KEY((“women soccer players” OR “women’s soccer” OR “female football players” OR “women’s football”) AND (“mental health” OR anxiety OR depression OR stress OR burnout OR “mental fatigue” OR wellbeing OR “psychological well-being” OR coping OR resilience OR “mental toughness”)).

Web of Science: TS = ((“women soccer players” OR “women’s soccer” OR “female football players” OR “women’s football”) AND (“mental health” OR anxiety OR depression OR stress OR burnout OR “mental fatigue” OR wellbeing OR “psychological well-being” OR coping OR resilience OR “mental toughness”)).

In the third stage, reference lists of the included articles were manually screened to identify additional studies not captured through electronic searches. All records were exported in comma-separated values (CSV) format and imported into Rayyan software (Rayyan Systems Massachusetts, USA, web 2026), where duplicates were removed. Title and abstract screening were conducted independently by two reviewers. Potentially eligible articles underwent full-text review. Discrepancies were resolved through consensus. The study selection process was reported in accordance with the Preferred Reporting Items for Systematic Reviews and Meta-Analyses extension for scoping reviews (PRISMA-ScR) checklist [31] (Supplementary Material), using a flow diagram detailing each stage of the screening process.

### 2.3. Eligibility Criteria

The eligibility criteria were established based on the PCC framework proposed by the JBI [30]. Inclusion criteria: (i) Studies that explicitly address mental health in women’s soccer players, participants competing at the semi-professional and professional levels, or in competitive settings for adolescents aged 13 to 18; (ii) studies evaluating psychological constructs related to mental health, including anxiety, mastery climate, depression, psychological stress, burnout, well-being, mental fatigue, resilience, coping strategies, self-confidence, satisfaction with sports, or psychological pressure; (iii) studies conducted in competitive soccer settings, including training sessions, official matches, preseason periods, tournaments, training camps, and club or national team environments; (iv) observational or experimental studies that evaluate the association, relationship, or predictive power of mental health or psychological factors, with performance as a secondary outcome, in female soccer players, regardless of their position on the field, provided that performance is assessed using a clearly defined and reproducible objective or subjective measure; (v) peer-reviewed articles with access to the full text; (vi) publications from January 2020 through December 2025. Exclusion criteria: (i) studies with insufficient data; (ii) studies focusing exclusively on male athletes; (iii) studies that included mixed populations without disaggregated data for women’s soccer players; (iv) studies in which women’s soccer is only part of a broad sports sample and it is not possible to identify specific results for variables related to psychological constructs and mental health; (v) letters to the editor, commentaries, protocols, conference abstracts, and documents without original results (Table 1).

These criteria were applied systematically and are reflected in the PRISMA-ScR flow diagram, which presents the number of records identified, screened, excluded, and included in the final review [31]. The PRISMA checklist was used to ensure transparency, accuracy, and quality in the publication of systematic reviews and meta-analyses.

### 2.4. Study Selection and Data Extraction

**Figure 1:**
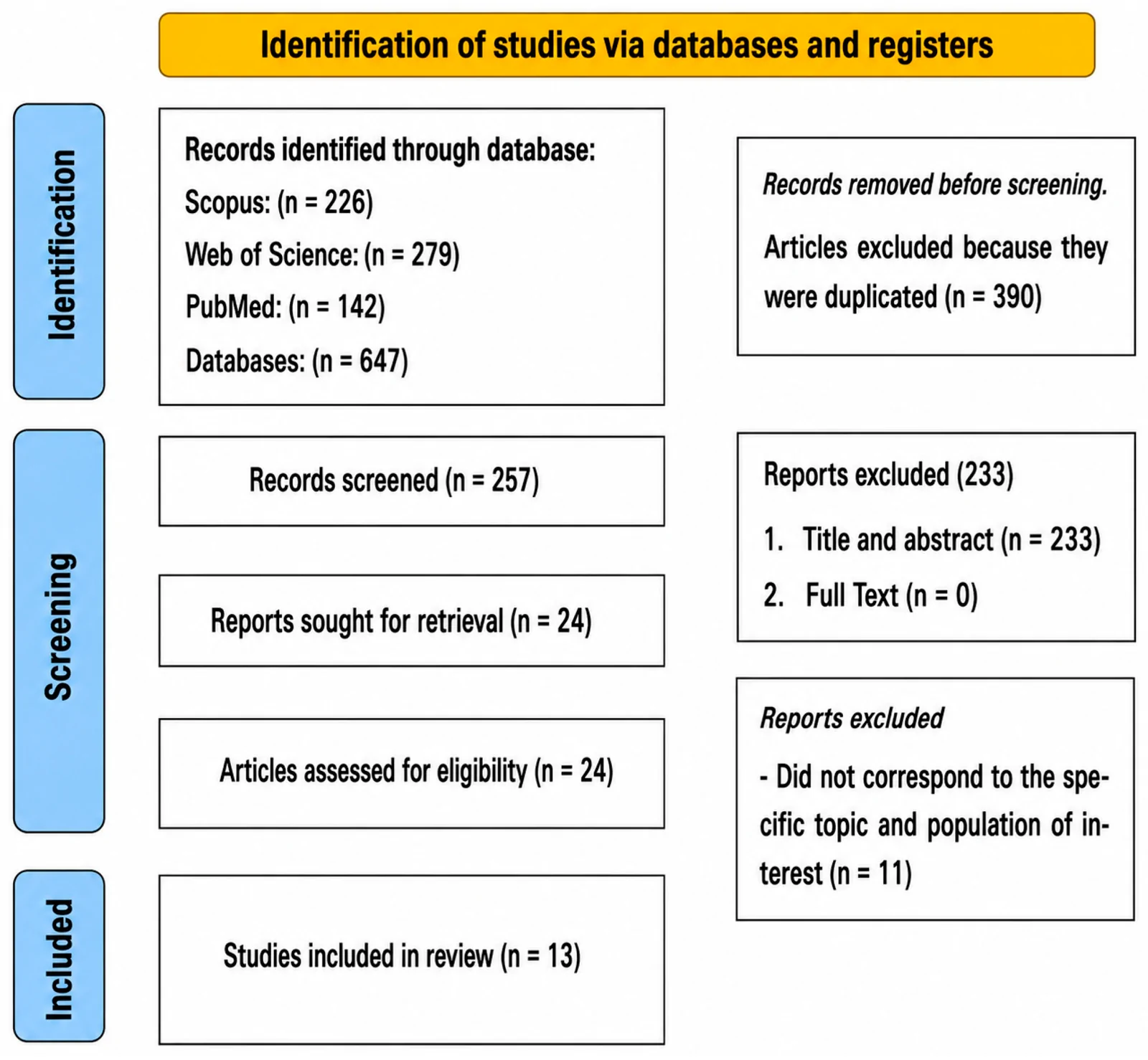
PRISMA Flow Diagram. Flow diagram illustrating the study selection process according to PRISMA-ScR guidelines, including the identification, screening, eligibility, and inclusion phases.

The identification phase across all databases yielded a total of 647 records. During the selection phase, 390 duplicate records were removed, leaving 257 records for review of titles and abstracts. Of these, 233 records were excluded at this stage, leaving 24 reports that were sought for retrieval. Articles for which the full text was not available were first searched for in institutional repositories and on ResearchGate; they were ultimately excluded after contacting the corresponding authors and receiving no response within four weeks. The evaluation of the full text led to the exclusion of 11 reports that did not align with the specific topic or the population of interest, in accordance with the predefined exclusion criteria. After applying all eligibility criteria, a total of 13 empirical studies were included in the final sample. Data extraction was performed independently by two reviewers. The variables extracted included: study design, sample size and characteristics, country, level of competition, assessment instruments, psychological variables assessed, and main outcomes. Discrepancies were resolved through discussion. The process of identifying and selecting the documents included in the review is illustrated in Figure 1.

## 3. Results

### 3.1. Characteristics of the Included Studies

Table 2 summarizes the main methodological, geographic, and temporal characteristics of the 13 studies included in the review. In terms of methodological design, longitudinal quantitative studies predominate (46.2%; *n* = 6), followed by cross-sectional quantitative studies (30.8%; *n* = 4), while qualitative studies account for 15.4% (*n* = 2) and mixed-methods studies for 7.7% (*n* = 1). Geographically, the evidence is concentrated primarily in Europe (76.9%; *n* = 10), with limited representation from South America (Brazil), Asia (Thailand), and Australia (7.7%; *n* = 1 each). Regarding the publication period, the largest proportion of studies corresponds to 2022–2023 (46.2%; *n* = 6), followed by 2020–2021 (30.8%; *n* = 4) and 2024–2025 (23.1%; *n* = 3). Overall, the results show evidence that is predominantly quantitative, longitudinal, and geographically concentrated in Europe, with an increase in publications during the intermediate period of 2022–2023.

Table 3 presents a summary of the main characteristics of the studies included in the scoping review. It describes the methodological design, sample characteristics, country of origin, and competitive level of the participating women’s soccer players. It also identifies the main measures and instruments used for assessment (Key Measures) and the variables or constructs analyzed (Variables Assessed), highlighting the diversity of approaches used to study mental health and psychological factors associated with competitive women’s soccer. Finally, the “Main Findings” column summarizes the key findings of each study, facilitating the identification of patterns related to anxiety, depression, psychological stress, burnout, resilience, coping, well-being, mood, recovery, and other relevant psychological and psychophysiological factors.

### 3.2. Mapping of Psychological Constructs and Instruments

The mapping of psychological constructs reveals a bi-dimensional research framework in the study of mental health in women’s soccer players. Specifically, the literature is concentrated on two main domains: (i) the measurement of psychological distress or psychopathology primarily stress, anxiety, and depression; (ii) measurement of positive and adaptive psychological resources, such as resilience, mental toughness, and motivation.

This dual methodological approach reflects a growing interest in understanding both vulnerability factors and internal resources that influence players’ mental health. The high frequency of mood-related variables (tension, anger, confusion) and anxiety suggest widespread use of standardized and validated instruments such as the Brunel Mood Scale (BRUMS) and Perceived Motivational Climate in Sport Questionnaire-2 (PMCSQ-2), facilitating comparability across studies. Additionally, the inclusion of constructs such as grit, perseverance and passion for long-term goals and personality traits indicates an emerging focus on stable psychological characteristics rather than transient emotional states (Table 4).

### 3.3. Outcome Measures: Psychological Factors Associated with Performance

The thirteen empirical studies included assessed various psychological variables, with an emphasis on anxiety, mood, and resilience. Ayuso-Moreno et al. [11] demonstrated that precompetitive anxiety is highly sensitive to the players’ experience and their status within the team; meanwhile, the qualitative evidence from Szabadics et al. [33] highlighted the contextual stressors experienced by professional women’s soccer players and suggested that resilience may function as a collective process rather than solely an individual one. Complementarily, other variables related to the team context included cohesion, competitive pressure, mental recovery, and satisfaction with the sport.

The empirical study by Pettersen et al. [34] identified a climate of mastery and extraversion as significant predictors of performance indicators; although this study did not directly assess mental health or well-being outcomes, its inclusion is important because it examined psychological resources such as perceived motivational climate, personality traits, self-regulation, and mental toughness. Taken together, these findings suggest that psychological factors have been studied predominantly in relation to performance or indicators of distress, while their specific role in the mental health and well-being of professional women’s soccer players is arguably less explored. This focus may contribute to and explain the existence of gaps in the integration of individual and contextual factors and in the understanding of positive psychological resources beyond their association with performance.

This trend is also evident in studies that focus directly on mental health indicators. Kilic et al. [16] assessed psychological distress, depression, anxiety, sleep disturbances, and disordered eating behaviors in soccer players in the Australian W-League, finding that 63% reported sport-related psychological distress and that greater resilience was associated with a 10% reduction in depressive symptoms. Ivarsson et al. [39], over an eight-week period during the COVID-19 pandemic with the AS Roma Serie A teams (mixed sample), reported that 36% of the women’s players met clinical criteria for depression at least once during the assessment period, along with higher levels of depression and negative affect among women. Taken together, these results reinforce the importance of anxiety, mood, and recovery aspects widely highlighted in previous reviews [5,41]. However, those reviews are syntheses of evidence rather than primary data sources; therefore, they are considered separately from the empirical evidence in this review. In this regard, the available primary evidence continues to present a fragmented picture, with greater emphasis on psychological symptoms and performance-related factors than on the interaction between well-being, psychological resources, and the sports context.

### 3.4. Mood States and Psychophysiological Markers

Throughout this section, a clear distinction is made between self-reported psychological measures, physiological biomarkers, and indicators of athletic performance, since these constructs should not be interpreted as interchangeable indicators of mental health.

Several studies integrated mood assessments with biomarkers of stress and fatigue. Botelho et al. [40] reported significant increases in negative mood states following preseason (*p* ≤ 0.03; effect size > 0.60), along with increases in salivary cortisol, testosterone, and creatine kinase (CK). Strong correlations were observed between cortisol and mood subscales (e.g., tension: Pearson’s correlation coefficient [r] = 0.53–0.60; confusion: r = 0.75), as well as a negative association between cortisol and heart rate variability low-frequency/high-frequency (LF/HF ratio: r = −0.52). These findings suggest that combined measures of mood (e.g., BRUMS) and biomarkers (cortisol, CK, heart rate variability) are sensitive tools for detecting stress responses during preseason in professional women’s soccer players.

### 3.5. Anxiety, Resilience and Coping

Anxiety was one of the most frequently studied variables, assessed in seven studies using validated instruments such as Sport Anxiety Scale-2 (SAS-2), Competitive State Anxiety Inventory-2 Revised (CSAI-2R), and Cognitive Appraisal of Training Questionnaire (CTA-Q). Madsen et al. [36] found that pre-match state anxiety was negatively associated with national team experience and positively associated with traits such as worry and fear of failure. Somatic anxiety was also linked to expectancy of success. Ayuso-Moreno et al. [11] reported a significant increase in cognitive anxiety prior to high-demand matches. Additionally, Perry et al. [37] reported a prevalence of moderate-to-severe anxiety of approximately 11%.

Bramley et al. [26] reported that 20.4% of semi-professional players exhibited anxiety symptoms, as assessed using the Generalized Anxiety Disorder-7 (GAD-7). Resilience was explicitly examined in two of the included studies, using markedly different approaches. Kilic et al. [16] measured it quantitatively using the Brief Resilience Scale (BRS) and found that higher resilience scores were associated with a 10% reduction in depressive symptoms, whereas Szabadics et al. [33] described team resilience as a collective process, involving shared coping strategies, learning from adversity, and coordinated responses to pressure.

This is consistent with the systematic review by Ventaja-Cruz et al. [3], which similarly found that resilience and coping strategies were associated with better adaptation and improved performance outcomes, although as a review-level synthesis it is not counted among the primary studies analyzed here. Bilgoe et al. [15] provided longitudinal evidence over 12 months showing that sport-related distress was the most prevalent mental health symptom (63%), and that sport-related distress increased almost twofold following surgery odds ratio (OR 2.0, 95% Confidence interval 0.9–3.8), underscoring the role of injury as both a physical and psychological risk factor. Despite its importance, heterogeneity in definitions and measurement approaches limit direct comparison across studies.

### 3.6. Mental Health and Symptom Prevalence

Bramley et al. [26] reported the following prevalence rates. It is important to note that the following prevalence figures are based on different instruments, cut-off points, and samples, and therefore are not directly comparable across studies. In a sample of 103 semi-professional players: psychological distress: 49.5%, depression: 44.7%, eating disorder symptoms: 22.3%, and anxiety: 20.4%. Similarly, Perry et al. [37] found that: moderate-to-severe depression 11.2%, moderate-to-severe anxiety 11%, and eating disorder symptoms 36%. These findings, based on specific samples of professional and semi-professional players, suggest a considerable burden of mental health symptoms in certain contexts of professional and semi-professional women’s soccer.

Additional convergent evidence from Kilic et al. [16] reported 63% sport-related distress among Australian W-League players; Ivarsson et al. [39] found 36% prevalence of clinical depression during the COVID-19 lockdown period in a sample of Italian Serie A players, with women’s soccer players consistently scoring higher than male players. It should be noted that these data were collected during an exceptional eight-week lockdown due to COVID-19, a situation that may not be comparable to a typical competitive season; and Bilgoe et al. [15] documented a 12-month prevalence of sport-related distress of 63%, depression of 13%, and eating disorder symptoms of 44% among professional women’s soccer players. Taken together, these data suggest that sports-related psychological distress may be the most common mental health problem in this population, as it consistently affects more than half of the players in various national contexts; however, this conclusion should be interpreted with caution given the heterogeneity and small number of studies. Consequently, these rates should not be interpreted as representative of women’s soccer.

### 3.7. Neurophysiological Measures and Performance

Tharawadeepimuk and Wongsawat [38] used quantitative electroencephalography (QEEG) to estimate activation levels and predict performance. They reported strong correlations between rapid perceptual responses and competitive performance (r ≈ 0.584; Spearman ρ ≈ 0.634; *p* < 0.001). High-performing players demonstrated lower anxiety and faster cognitive responses to stimuli. Additionally, factors such as central fatigue and assertiveness appeared to influence performance and could differentiate starting players from substitutes.

### 3.8. Lifestyle, Sleep and Recovery

Fältström et al. [35] examined lifestyle indicators, reporting that: 32% of players experienced frequent stress, and 24% were classified with psychological distress. Significant increases were observed in medication use, meal skipping, and fatigue over a one-year period (*p* = 0.031 to <0.001). In addition, Thompson et al. [32], using focus groups, identified mental fatigue sources such as travel demands, academic/work obligations, and disruptions to daily routines. Players also reported positive effects of music before competition and highlighted discomfort associated with prolonged exposure to soccer-related activities. These findings suggest that stress, fatigue, and daily habits are closely linked to the demands of athletic training, highlighting the importance of considering lifestyle and recovery conditions beyond strictly competitive demands.

### 3.9. Methodological Approaches

Three dominant methodological approaches were identified:

1. Quantitative designs, including surveys, biomarkers, and QEEG.
2. Qualitative designs, including interviews, and thematic analysis.
3. Mixed methods approach.

Quantitative analyses typically included descriptive statistics, correlations, regression models, and effect size estimation. Qualitative studies used reflexive thematic analysis. Some studies demonstrated the value of integrating subjective and objective measures, reducing bias associated with self-report data.

### 3.10. Future Research Directions Identified from the Synthesis

The synthesis of the included studies highlights several key directions for future research, including the need to:

- Expand the use of longitudinal designs, as this type of design allows for the observation of the actual evolution of symptoms and psychological resources throughout competitive seasons.
- Integrate multi-method approaches, as only one study combined quantitative and qualitative data.
- Investigating contextual and environmental factors specific to Latin America and Asia, regions that together represent approximately 15% of the included studies (one study each from South America and Asia).
- Develop intervention-based research, given that none of the included studies evaluated a psychological intervention.

**Figure 2:**
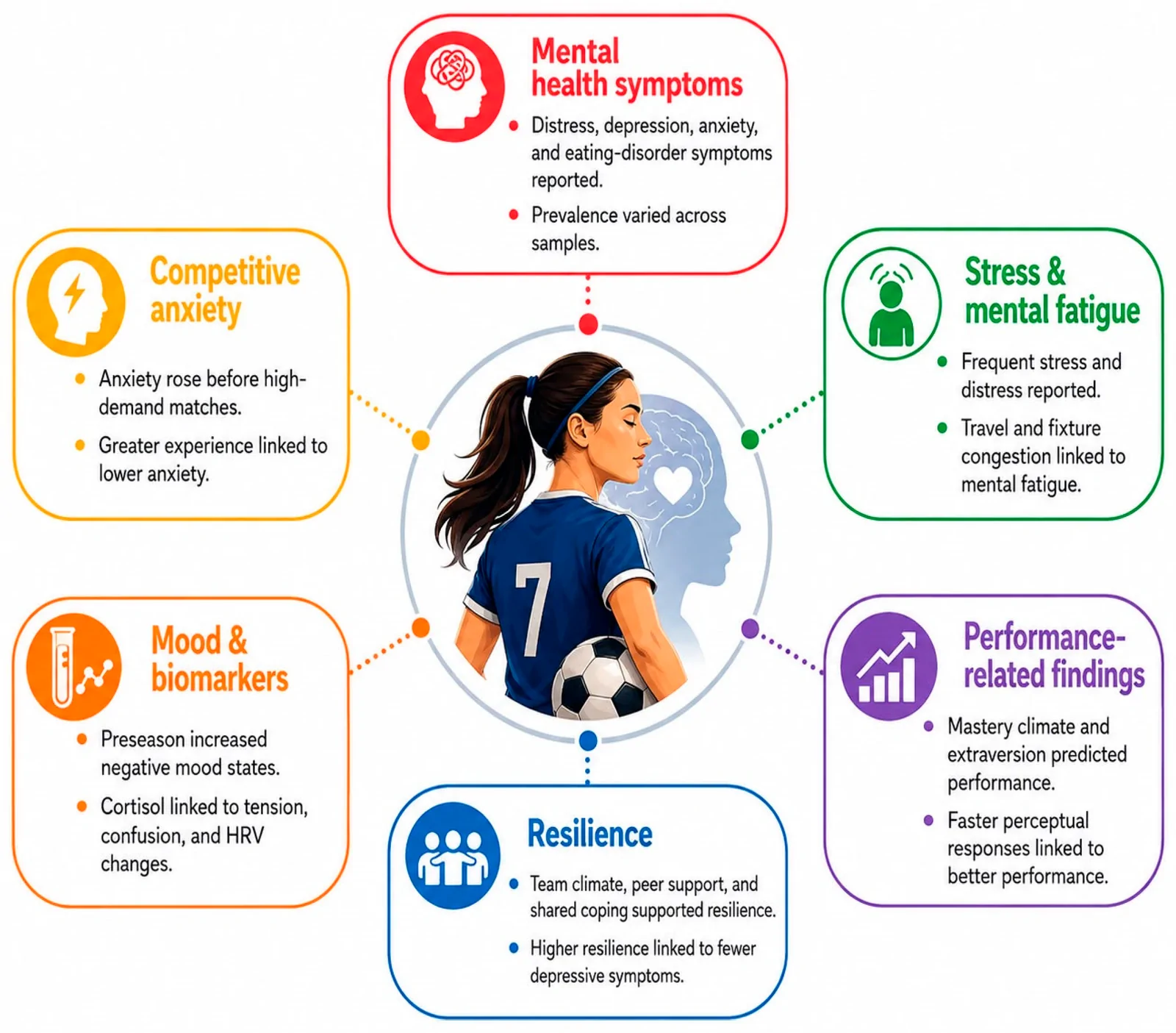
Key considerations for future research on mental health in competitive adolescents, semi-professional and professional women’s soccer players.

These directions are summarized in Figure 2.

## 4. Discussion

The findings of this scoping review demonstrate a notable increase in research on mental health in women’s soccer players over the past five years, although the evidence remains geographically uneven, with a predominance of European studies. The literature reviewed consistently suggests that research has been structured around two complementary domains: negative psychological states (particularly anxiety, stress, and depressed mood) and adaptive psychological resources such as resilience and coping mechanisms. This duality, previously highlighted in systematic reviews [7], appears to represent the conceptual foundation of contemporary psychological research in women’s soccer.

Ayuso-Moreno et al. [11] demonstrated that cognitive anxiety increases significantly prior to important matches and is modulated by competitive status and player experience. These findings align with qualitative research describing perceptions of pressure and fear of failure as central stressors within professional environments [33].

Although this review did not include studies that directly assessed the relationship between media exposure and emotional vulnerability in women’s soccer, it could be suggested that factors such as media exposure, external expectations, and constant comparison with men’s leagues contribute to such vulnerability. Nevertheless, this hypothesis opens an interesting avenue for future research in this field, which could analyze the effect of media exposure, external expectations, and constant comparison with men’s leagues on emotional vulnerability, in order to understand the influence these psychological constructs have in various broader sociocultural and sports contexts, as well as in contexts specific to women’s soccer.

Stress also emerges as a central component of mental health in this population. Botelho et al. [40], using psychophysiological markers such as salivary cortisol, showed that players experience significant stress elevations during preseason, accompanied by notable changes in mood profiles. The convergence of physiological and psychological indicators reinforces the notion that mental health in professional athletes should not be addressed in isolation, but rather as part of an integrated system involving physical load, recovery quality, and contextual demands associated with competitive calendars [41].

In contrast to the extensive focus on psychological distress, fewer studies have examined adaptive resources such as resilience, motivation, and mental toughness, although these provide important conceptual contributions. It should be noted that the conceptualization of resilience varied considerably among the included studies, ranging from an individual trait-based approach to a collective process dependent on the team context, which makes it difficult to directly compare the results. Similarly, motivation, mental toughness, coping ability, and well-being were not always clearly distinguished at the conceptual level across the different studies. Furthermore, Ventaja-Cruz et al. [3] noted that resilience was associated not only with lower levels of perceived stress but also with better individual and collective performance outcomes, although this conclusion is based on a small number of studies.

Complementarily, Szabadics et al. [33] proposed a novel perspective, suggesting that resilience in women’s soccer operates primarily as a collective phenomenon emerging from team climate, peer support, and leadership quality. This collective perspective contrasts with traditional approaches centered on individual traits and represents a promising emerging direction for research. It is important to note that the included studies share common methodological limitations, such as small sample sizes, reliance on self-report instruments, limited geographic representation outside Europe, and considerable heterogeneity in the psychological measures used, which limits the strength of the conclusions that can be drawn from the current evidence.

Another relevant aspect concerns the role of stable psychological traits and long-term competencies. Pettersen et al. [34] identified that variables such as self-confidence, self-regulated learning, emotional stability, and perseverance significantly contribute to performance outcomes, beyond physical or tactical factors. It bears repeating that these performance-related constructs are interpreted here solely in relation to mental health and not as equivalents to it in line with the conceptual distinction established in the introduction. This emphasis on stable characteristics, combined with the incorporation of innovative methodologies such as QEEG [38], suggests that research is moving toward more integrative models that aim to understand how players perceive, process, and regulate behavior under high-demand conditions.

Despite these advances, the evidence reveals some significant gaps. First, it should be clarified that, although quantitative longitudinal studies constituted the most common individual methodological category at 46.2%, these figures should not be interpreted as evidence of methodological maturity: more than half of the included studies continued to rely on cross-sectional or single-time-point designs, and even the longitudinal studies identified were limited to short observation windows (for example, an eight-week COVID-19 lockdown period), none of which tracked the players over the course of several full competitive seasons. Second, there is limited representation of professional leagues outside Europe, which restricts the generalizability of findings and overlooks sociocultural contexts where structural inequalities may further influence mental health outcomes. Additionally, there is insufficient research addressing specific issues such as body image, eating disorders, and mental health during injury and return-to-play phases, despite their documented relevance in professional sport populations. These gaps highlight the need for more comprehensive and context-sensitive approaches.

It is worth situating these findings along a recently published scoping review by Perry et al. [42], which mapped mental ill-health symptoms (depression, anxiety, and eating disorder symptoms) and their competition-related risk factors across ten exclusively quantitative studies on professional women’s soccer. It should be noted that this review was published after the final search date for this study and, therefore, could not be identified through the systematic search strategy; it was included solely for the purpose of contextual comparison. While the two reviews share a common interest in mental health within professional women’s soccer, their scope and conclusions diverge in important ways. Perry et al. [42] explicitly identifies the exclusive reliance on quantitative, self-report designs as a major limitation of the existing evidence base, noting that this has hindered understanding of players’ lived experiences and of broader socio-cultural stressors such as contract instability, social media exposure, body image, motherhood, and discrimination. The present review addresses part of this gap by deliberately integrating qualitative evidence, psychophysiological and neurophysiological biomarkers, and adaptive psychological resources (resilience, coping, motivation) alongside symptom-level data, and by extending the population beyond professional players to semi-professional and adolescent competitive contexts. Rather than duplicating the work of Perry et al. [42], this review therefore offers a complementary, broader bio-psychosocial mapping of the field, reinforcing from an independent angle their call for more methodologically diverse research.

From an applied perspective, these findings suggest the need to incorporate multidisciplinary support teams (sports psychologists, medical staff, and coaching staff) into the structures of women’s soccer clubs. Routine psychological monitoring systems, like the Sport Mental Health Assessment Tool-1 (SMHAT-1) used in two of the included studies, could facilitate the early detection of symptoms of distress. Likewise, organizational policies that address travel burden, schedule congestion, and support during injury and return-to-play processes could mitigate risk factors identified in this review.

Unlike Perry et al. [42], which included 10 exclusively quantitative studies, this review covers the period from 2020 to 2025 in the selected databases and incorporates qualitative evidence, semi-professional populations, and adolescents. It should be noted that the findings are not fully consistent across studies; while Bilgoe et al. [15] and Kilic et al. [16] reported sports-related distress exceeding 60%, Perry et al. [37] reported considerably lower rates of depression and anxiety (11–11.2%), which could reflect differences in the instruments used, diagnostic cut-off points, or sample characteristics.

Overall, the findings suggest that mental health in women’s soccer should be conceptualized within a multifactorial framework that integrates physiological load, contextual demands, psychological perceptions, adaptive competencies, and team dynamics. The evidence suggests a strong interdependence between psychological well-being and athletic performance, emphasizing the need for intervention strategies that address both individual emotional regulation and organizational support systems within clubs and professional structures.

### 4.1. Limitations

This scoping review is subject to several methodological and contextual limitations. First, the restriction of the search period to five years, from 2020 to 2025, although useful for capturing recent evidence, may have excluded earlier foundational studies.

Second, the search was limited to PubMed/MEDLINE, Scopus, and Web of Science, and did not include PsycINFO or any other database specializing in psychology; since this review addresses constructs in mental health and sports psychology, this omission may have excluded relevant literature indexed in psychology that is not covered by the three databases consulted; therefore, future reviews in this field should incorporate PsycINFO or an equivalent specialized database. Third, the exclusion of studies due to lack of open-access full texts may have limited the comprehensiveness of the evidence base and potentially omitted relevant high-impact research. A dual contextual bias was identified in the mapped literature: first, a pronounced geographic bias, with most studies conducted in European high-performance leagues, limiting the generalizability of findings to other regions, such as Latin America and Asia; and second, a general underrepresentation of the topic, as reflected in the relatively small number of included empirical studies (*n* = 13), suggesting that mental health in professional women’s soccer remains an underexplored research area. The conceptual heterogeneity among the included studies, as well as the variation in the diagnostic instruments and cut-off points used, limits the direct comparability of the findings. The inclusion of different levels of competition and, in one case, a mixed-sex sample, introduces additional heterogeneity into the evidence base. The failure to conduct a formal assessment of the methodological quality of the included studies could be a limitation, which may hinder the interpretation of the results; therefore, it is suggested that future reviews particularly systematic reviews incorporate this process.

### 4.2. Future Perspectives

Future research directions emerge directly from the methodological and contextual gaps identified in this review. From a methodological perspective, there is a clear need to prioritize longitudinal study designs that allow researchers to track psychological constructs over time and facilitate stronger causal inference. Additionally, the evaluation of psychological interventions through controlled trials is essential to establish their effectiveness in this population. In terms of contextual and population diversity, there is an urgent need to expand research to underrepresented regions, particularly in Latin American leagues, to better understand the influence of cultural and institutional factors on mental health outcomes. Furthermore, future studies should explore mental health in specific playing positions, such as goalkeepers, as different roles within the team may involve distinct psychological demands. Regarding the conceptual framework, future research should move beyond a predominant focus on psychopathology and incorporate a stronger emphasis on positive mental health and well-being, supporting sustainable performance and holistic athlete development across all competitive levels.

## 5. Conclusions

This scoping review provides a specific overview of recent evidence from scientific literature on the mental health of professional, semi-professional and adolescent competitive women’s soccer players between 2020 and 2025.

The findings suggest that research in this field has primarily concentrated on competitive anxiety, psychological stress, and mood state profiles, establishing these variables as the most consolidated areas of study.

At the same time, there is a growing interest in positive psychological resources (particularly resilience, mental toughness, and coping strategies) which contribute to a more integrative understanding of players’ emotional experiences under high-performance conditions. These conclusions should be interpreted as specific to the small and heterogeneous set of studies included, and not as representative of women’s soccer. It should be noted that this review maps the available evidence, but its design does not allow for the establishment of causal or integrated relationships among the identified physiological, psychological, and contextual factors.

The included studies suggest that psychological distress and adaptive resources coexist as two fundamental dimensions of the psychological experience in women’s soccer. However, current evidence tends to emphasize the measurement of vulnerabilities rather than the systematic analysis of protective factors.

Furthermore, there is a gradual incorporation of innovative methodologies, such as physiological biomarkers and neurophysiological technologies, which expand assessment possibilities beyond self-report measures. Despite these advances, the field remains limited by the fact that more than half of the studies continued to rely on cross-sectional or single-time-point designs, the underrepresentation of regions outside Europe, and the short observation periods of the identified longitudinal studies, which restrict the establishment of multi-seasonal and multi-component frameworks. Overall, these findings highlight the need to address mental health in women’s soccer through a contextualized and multidimensional approach, considering not only individual factors but also team dynamics, competitive demands, and the structural characteristics of the sporting environment.

## Figures and Tables

**Table 1:** Inclusion and exclusion criteria.

Inclusion	Exclusion
Women’s soccer players competing at semi-professional, professional and adolescent competitive levels	Recreational or amateur participants without formal competition
Explicit assessment of at least one mental health variable: anxiety, depression, psychological stress, burnout, resilience, well-being, coping strategies, self-confidence, sport satisfaction	Studies in which mental health is not assessed or is only addressed superficially
Quantitative, qualitative, or mixed-method studies	Reviews, opinion articles, editorials, non-empirical studies, conference proceedings, grey literature
Original research articles with full-text access	Articles without full-text access or insufficient data
Published between 2020 and 2025	Publications prior to 2020
Studies conducted in competitive soccer contexts (training, matches, preseason, leagues, national teams)	Studies in other sports or mixed samples without women-specific data

**Table 2:** General characteristics of the included studies (*n* = 13).

Category	Specification	Frequency (*n*)	Percentage (%)
Methodological design	Quantitative (longitudinal)	6	46.2%
	Quantitative (cross-sectional)	4	30.8%
	Qualitative	2	15.4%
	Mixed-methods	1	7.7%
Geographic location	Europe	10	76.9%
	South America (Brazil)	1	7.7%
	Asia (Thailand)	1	7.7%
	Australia	1	7.7%
Publication period	2020–2021	4	30.8%
	2022–2023	6	46.2%
	2024–2025	3	23.1%

Note: *n*: number; %: percentage.

**Table 3:** Characteristics of the 13 included empirical studies (study design, sample, country, competition level, outcome measures, variables, and main findings).

Reference	Study Design	Sample	Country	Competition Level	Key Measures	Variables Assessed	Main Findings
Ayuso-Moreno et al. [11]	Quantitative	*n* = 14	Spain	Semi-professional	CSAI-2R; Polar RS800CX (HRV)	Competitive anxiety; heart rate variability	High-demand matches increased cognitive anxiety and reduced HRV (LF/HF ratio); somatic anxiety and HRV showed similar pattern
Bilgoe et al. [15]	Quantitative longitudinal (12-month follow-up)	*n* = 74 women professionals (Eredivisie Vrouwen)	Netherlands	Professional	SMHAT-1 (annual application); validated subscales for distress, depression, anxiety, eating disorders	Psychological distress, depression, anxiety, sleep, disordered eating, alcohol misuse; injury as risk factor	Distress 63%, depression 13%, disordered eating 44%; associated with higher odds of sport-related distress after surgery (OR 2.0, 95% CI 0.9–3.8); first 12-month longitudinal study exclusively in women professional players
Kilic et al. [16]	Quantitative cross-sectional	*n* = 132 (W-League)	Australia	Professional	SMHAT-1; GHQ-12; K-10; AUDIT; BEDA-Q; BRS (resilience)	Psychological distress, depression, anxiety, sleep, disordered eating, alcohol misuse, resilience	63% sport-related distress in women players; injury → greater sleep disturbance; higher resilience associated with 10% reduction in depressive symptoms
Bramley et al. [26]	Mixed-methods (pragmatic)	*n* = 103	England	Semi-professional	K-10; CES-D; GAD-7; BEDA-Q; semi-structured interviews	Distress, depression, anxiety, eating disorders	49.5% distress; 44.7% depression; 20.4% anxiety; 22.3% eating disorder risk. Lifestyle factors (medication, skipping meals) increased
Thompson et al. [32]	Qualitative (focus groups)	*n* = 10	England	Professional	Qualitative interview schedule	Mental fatigue, stress, travel burden	Perceived travel demands, combined with fixture congestion, emerge as primary sources of mental fatigue
Szabadics et al. [33]	Qualitative (critical realism)	*n* = 27	England	Professional	Interviews; focus groups	Team resilience; collective coping	Resilience operates as a collective process shaped by team climate, peer support, and leadership quality
Pettersen et al. [34]	Quantitative cross-sectional	*n* = 156	Norway	Professional (top-2 leagues)	PMCSQ-2; BFI-20; SMTQ	Motivational climate; personality; self-regulation	Mastery climate was a significant predictor of performance in competition. Soccer players who perceived a team environment that was more focused on learning, improvement, effort, and development demonstrated better individual performance in matches.
Fältström et al. [35]	Quantitative longitudinal	*n* = 419	Sweden	Adolescent competitive	GHQ-12; Brief COPE; PSQI; IPAQ	Anxiety, depressed mood, social function, sleep	Older, more experienced players had lower trait anxiety; 16% reported sleep <8 h; supplement and screen use prevalent; 32% experience stress, and 24% experience psychological distress.
Madsen et al. [36]	Quantitative longitudinal	*n* = 180	Denmark	Professional	AMS-S; SAS-2; CSAI-2R; CTA-Q	Pre-competitive anxiety; worry; fear of failure	National team experience predicted lower state anxiety; somatic anxiety linked to expectancy of success; worry predicts cognitive anxiety
Perry et al. [37]	Quantitative cross-sectional	*n* = 115	England	Professional (WSL/Championship)	PHQ-9; GAD-7; BEDA-Q; GHSQ	Depression, anxiety, eating disorders, help-seeking	11.2% moderate–severe depression; 11% moderate–severe anxiety; 36% eating disorder risk; 73% had not sought help
Tharawadeepimuk and Wongsawat [38]	Quantitative electroencephalography (QEEG)	*n* = 32	Thailand	Professional (national team)	QEEG; SAS-2	Anxiety; neurophysiological performance indicators	Faster perceptual responses linked to superior performance (r = 0.584); lower anxiety combined with optimal cortical activation predicts higher performance
Ivarsson et al. [39]	Longitudinal survey (8-week COVID-19 lockdown)	*n* = 101	Italy	Professional (Serie A)	CES-D; PANAS; BRUMS (4 time points)	Depression, mood states, positive/negative affect, psychological well-being	36% reported clinical depression at ≥1 time point; depressive symptoms decreased over 8 weeks; women’s soccer players reported higher depression and negative affect than male players
Botelho et al. [40]	Quantitative observational-descriptive	*n* = 24	Brazil	Professional	BRUMS; cortisol, testosterone, CK	Mood states; psychophysiological markers	Preseason increased negative mood states; cortisol positively correlated with tension (r = 0.53–0.60) and confusion (r = 0.75)

Note: BRUMS: Brunel Mood Scale; CK: creatine kinase; K-10: Kessler Psychological Distress Scale; CES-D: Center for Epidemiologic Studies Depression Scale; GAD-7: Generalized Anxiety Disorder-7; PMCSQ-2: Perceived Motivational Climate in Sport Questionnaire-2; LF/HF: low-frequency/high-frequency; BEDA-Q: Brief Eating Disorder Questionnaire; GHQ-12: General Health Questionnaire-12; AUDIT: Alcohol Use Disorders Identification Test; SMTQ: Sport Mental Toughness Questionnaire; BRS: Brief Resilience Scale; SMHAT-1: Sport Mental Health Assessment Tool-1; CSAI-2R: Competitive State Anxiety Inventory-2 Revised; AMS-S: Achievement Motive Scale—Sport; SAS-2: Sport Anxiety Scale-2; CTA-Q: Cognitive Appraisal of Training Questionnaire; PANAS: Positive and Negative Affect Schedule; PSQI: Pittsburgh Sleep Quality Index; IPAQ: International Physical Activity Questionnaire; PHQ-9: Patient Health Questionnaire-9; HRV: Heart rate variability; QEEG: Quantitative electroencephalography; GHSQ: General Help-Seeking Questionnaire; COVID-19: Coronavirus Disease 2019; WSL: Women’s Super League; OR: odds ratio; CI: confidence interval.

**Table 4:** Psychological constructs and instruments.

General Psychological Construct	Specific Variables Identified	Typical Measurement Instruments	Key Findings
Psychological distress and negative affect	Tension, depression, anger, fatigue, confusion; anxiety; distress; depressed mood; neuroticism	BRUMS; GHQ-12; SMHAT-1	High prevalence of negative emotional states in competitive contexts
Adaptive resources and resilience	Resilience; mental toughness; determination; recovery; grit	Coping scales; GHSQ	Associated with better adaptation and performance
Sport-specific anxiety	Cognitive anxiety; somatic anxiety; fear of failure; concern	SAS-2; CSAI-2R; CTA-Q	Sensitive to competition level and experience
Social and performance constructs	Cohesion; motivation; passion; social function	PMCSQ-2	Linked to performance and well-being
Personality traits	Openness, conscientiousness, extraversion, agreeableness	BFI-20	Emerging predictors of performance
Other	Eating disorders	BEDA-Q	Understudied but relevant

Note: BRUMS: Brunel Mood Scale; GHQ-12: General Health Questionnaire; GHSQ: General Help-Seeking Questionnaire; SAS-2: Sport Anxiety Scale-2; CSAI-2R: Competitive State Anxiety Inventory-2 Revised; CTA-Q: Cognitive Appraisal of Training Questionnaire; PMCSQ-2: Perceived Motivational Climate in Sport Questionnaire-2; BFI-20: Big Five Inventory; BEDA-Q: Brief Eating Disorder Questionnaire; SMHAT-1: Sport Mental Health Assessment Tool-1.

## Data Availability

The original contributions presented in this study are included in the article/Supplementary Material. Further inquiries can be directed to the corresponding author.

## Supplementary Materials

The following supporting information can be downloaded at: https://www.mdpi.com/article/10.3390/sports14090390/s1, Reference [43] are cited in the supplementary materials.
